# Pancreatic Heterotopia Presenting as a Mimic of Fistulizing Crohn’s Disease: Expanding the Differential Diagnosis of Inflammatory Bowel Disease

**DOI:** 10.7759/cureus.68666

**Published:** 2024-09-04

**Authors:** Yeseena Alli, Jesus S Noain, Wei Shaw, Kelly Brenan

**Affiliations:** 1 Gastroenterology, Drexel University College of Medicine, Philadelphia, USA; 2 Gastroenterology, Tower Health Reading Hospital, West Reading, USA; 3 Radiology, Tower Health Reading Hospital, West Reading, USA; 4 Pathology, Tower Health Reading Hospital, West Reading, USA

**Keywords:** ectopic pancreas, jejunal mass, inflammatory bowel disease, crohn’s disease (cd), heterotopic pancreas

## Abstract

Pancreatic heterotopia (PH) involves pancreatic tissue located outside its typical anatomical position, lacking vascular or ductal communication with the pancreas. Despite frequently having acini with the capacity to produce digestive enzymes, PH is usually asymptomatic. When symptoms do occur, they typically present in middle to late adulthood and include abdominal pain, nausea, and diarrhea. This clinical presentation is similar to that of Crohn’s disease, an autoimmune inflammatory bowel disease (IBD). The presentation of symptomatic PH varies depending on the location of the ectopic pancreatic tissue and its microanatomical constituents, including exocrine and endocrine tissue as well as a duct system. We present a case of a patient who came to medical attention with abdominal pain and was found on colonoscopy to have a non-obstructing stricture of the transverse colon without an associated mass. Biopsies of the area revealed chronic active colitis, leading to a diagnosis of Crohn’s disease. Her gastroenterological symptoms remained stable for several years while receiving infliximab infusions until she presented to the emergency department with severe abdominal pain, diarrhea, and sepsis, meeting the criteria for systemic inflammatory response syndrome. Imaging studies revealed a fistula between the previous colonic stricture and the jejunum, again attributed to Crohn’s disease. She underwent surgery to remove the fistula between the small and large bowels. Unexpectedly, the resection specimen showed a mass insinuated between the loops of the large intestine, which histological review revealed to be ectopic pancreatic tissue. Following the resection of the ectopic pancreatic tissue, her symptoms resolved without the need for further treatment. In retrospect, the ectopic pancreatic tissue, which contained acini with digestive enzymes, ducts, and islets, may have also caused seemingly unrelated pathology in the patient. Symptomatic PH should be recognized as a pathology that can mimic IBD, prompting reconsideration of the diagnosis in cases of refractory disease while on biologics.

## Introduction

Pancreatic heterotopia (PH) refers to pancreatic tissue that lacks vascular or anatomical communication with the normal pancreas. Despite being present in a significant portion of the population (a reported incidence of 0.11-13%, depending on the study cited), it is rarely symptomatic [1]. If symptoms do emerge, they manifest as non-specific symptoms, including abdominal pain, nausea, and diarrhea [1]. Crohn’s disease is an autoimmune inflammatory bowel disease (IBD) that has a similar incidence (0.3% of the US population) but is generally symptomatic in affected patients [2]. Crohn’s disease presents clinically with chronic diarrhea, hematochezia, weight loss, and abdominal pain with signs of large or small bowel inflammation on imaging and/or endoscopy [3]. There are different pathologic findings in Crohn’s disease, which include inflammatory, stricturing, and penetrating (or fistulizing) disease [4]. Often patients progress from inflammatory Crohn’s disease to stricturing or penetrating disease. Typically, biopsies of affected mucosa may show acute-on-chronic inflammation with or without granulomas. Here, we present a case of a patient with medical presentation, imaging, and histopathological findings that mimic those of IBD caused by ectopic pancreatic tissue in the jejunum. Following resection of the ectopic pancreatic tissue, the patient’s symptoms of IBD abated without further treatment.

## Case presentation

**Figure 1:**
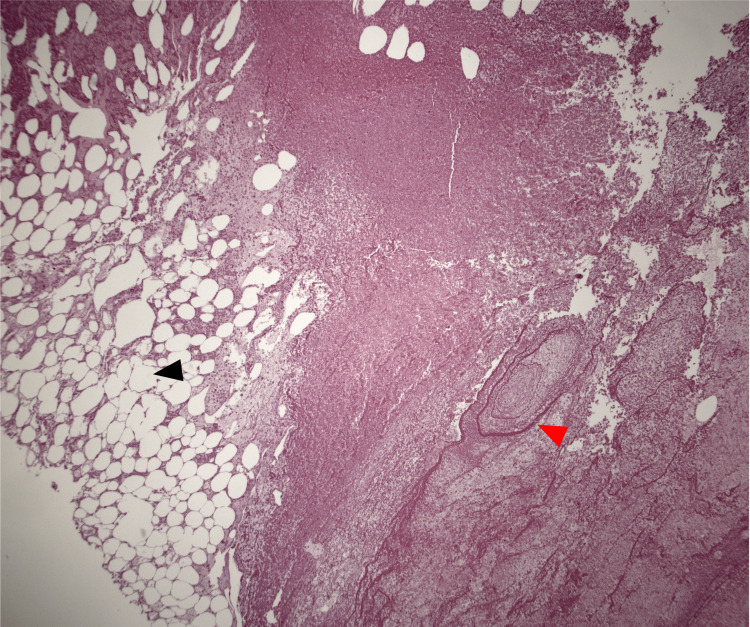
Adrenal bed demonstrating significant fat saponification (red arrowhead) compared to preserved fat cells (black arrowhead), which is postulated to be due to pancreatic enzyme being released into the peritoneal cavity (low power (2x), H&E) H&E: hematoxylin and eosin

**Figure 2:**
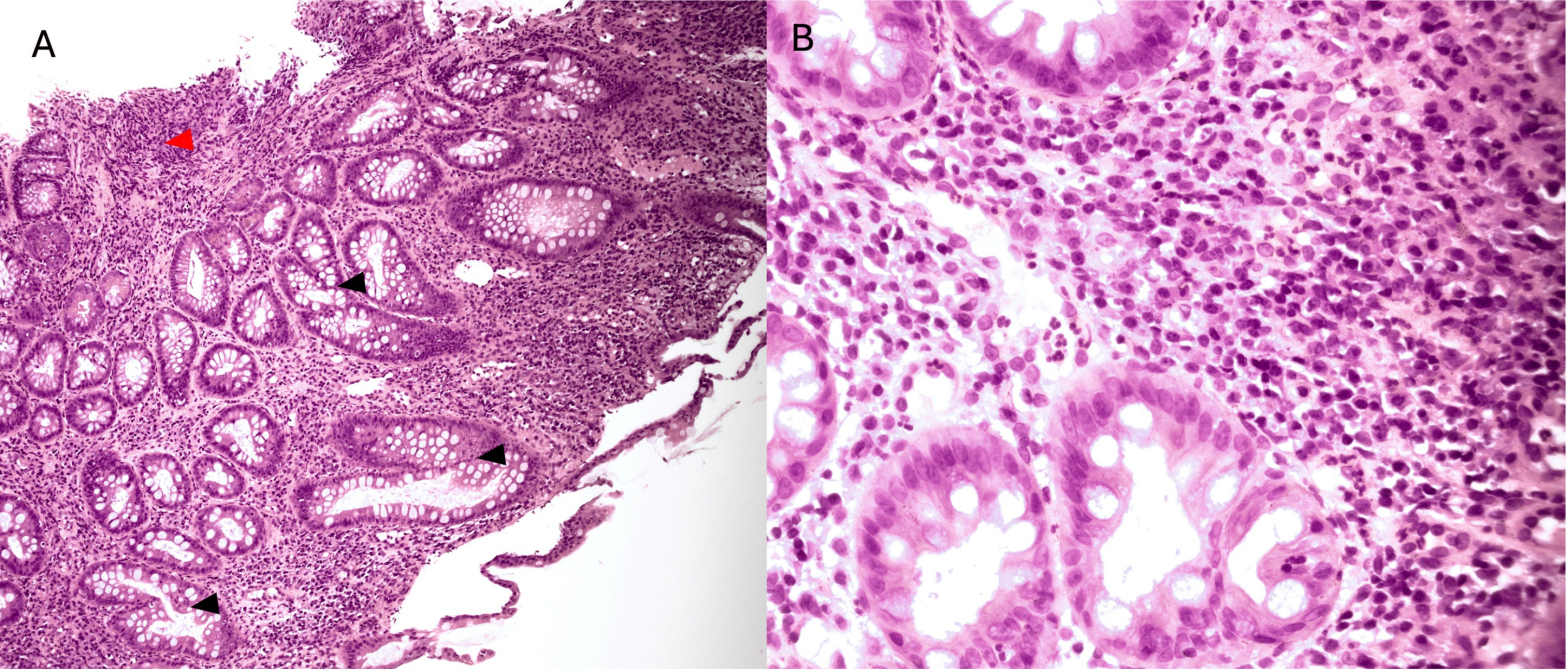
Evidence of acute-on-chronic colitis (A: low power (2x), H&E; B: medium power (10x), H&E) (A) Low-power magnification of the colon revealing architectural distortion with crypt loss (red arrowhead) and regenerative crypt changes, along with multiple areas of crypt fusion (black arrowheads). (B) Initial colon biopsies depict architectural distortion with crypt loss and neutrophilic infiltrate characteristic of chronic active colitis suggestive of IBD, such as Crohn's disease. H&E: hematoxylin and eosin, IBD: inflammatory bowel disease

A 57-year-old female with a medical history significant for a myelolipoma of the left adrenal gland, status post-adrenalectomy, and poor healing of the surgical bed, as well as a gastroenterological history marked by a transverse colon stricture discovered on colonoscopy (Figure 1), presents for evaluation. At the time of the procedure, the patient had mild abdominal pain, and the endoscopy revealed friable and ulcerated mucosa of the transverse colon, along with an anal fistula. Biopsies of the transverse colon stricture showed mild acute-on-chronic colitis, interpreted to be consistent with Crohn’s disease (Figure 2A-2B). Endoscopic examination of the terminal ileum showed no evidence of disease, and biopsies confirmed the absence of pathological abnormalities.

**Figure 3:**
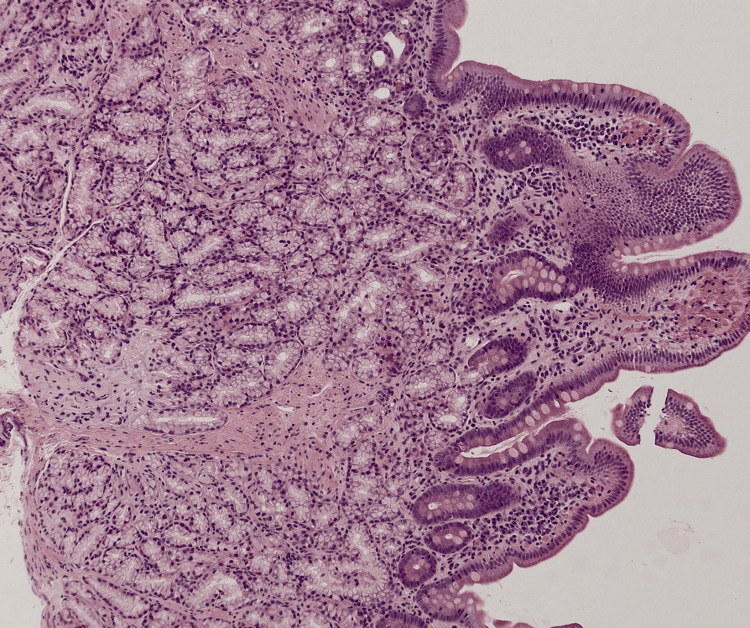
Enlarged Brunner's glands in a location closer to the lumen of the small intestine, suggesting chronic duodenitis (low power (2x), H&E) H&E: hematoxylin and eosin

Several months later, repeat biopsies of the transverse colon demonstrated moderate active colitis, again interpreted to be consistent with the Crohn’s diagnosis. Samples from an upper endoscopy were remarkable for Brunner’s gland hyperplasia in the duodenum (Figure 3). She was treated with Infliximab and followed by gastroenterology.

**Figure 4:**
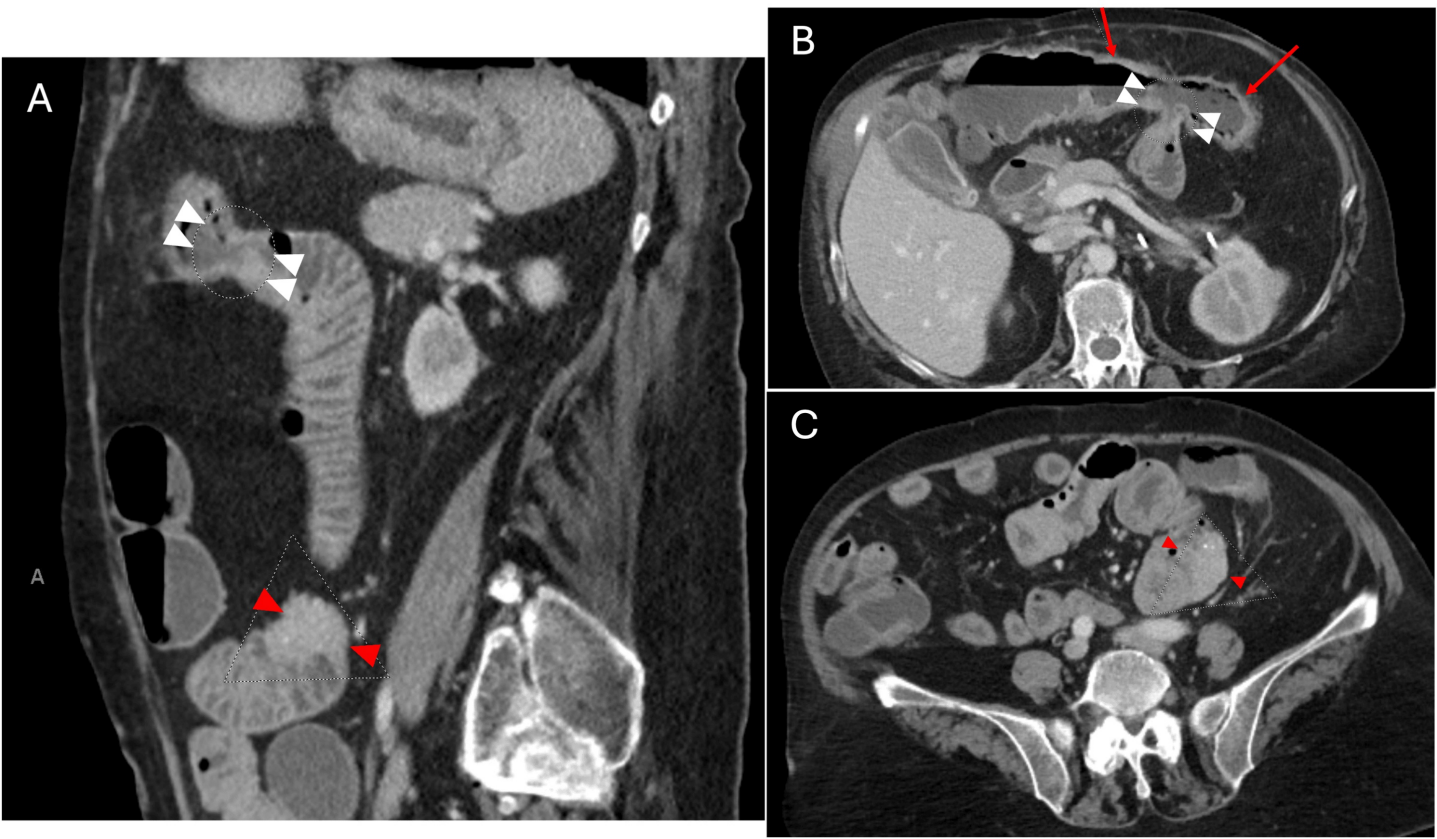
CT abdomen and pelvis (A) Sagittal view demonstrating evidence of a fistula (white arrowheads) and an irregularly margined lobular enhancing mass (red arrowheads) distal to the fistula. (B) Axial view depicting the fistula (white arrowheads) between the transverse colon (red arrows) and proximal jejunum, with mild colonic wall thickening and mild adjacent fat stranding. (C) Axial view illustrating the lobular enhanced mass (red arrowheads) invading the lateral aspect of the proximal jejunum. CT: computed tomography

**Figure 5:**
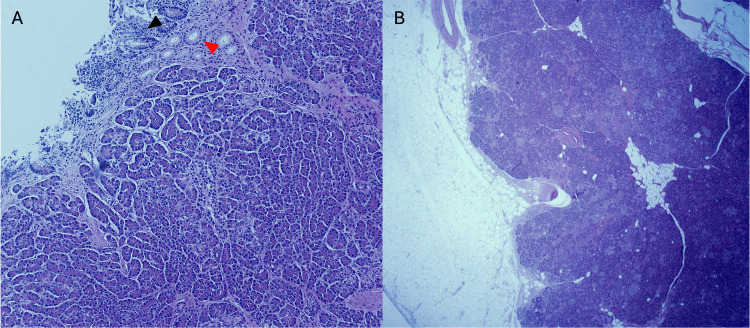
Local invasion of ectopic pancreatic tissue (A: low power (4x), H&E; B: low power (2x), H&E) (A) Small bowel mucosa with areas of denudation and focal remaining glands (black arrowhead) with heterotopic pancreatic ducts (red arrowhead) and acini extending into the lamina propria of small bowel mucosa. (B) Evidence of heterotopic pancreatic tissue extending from the bowel wall into the pericolic fat. H&E: hematoxylin and eosin

**Figure 6:**
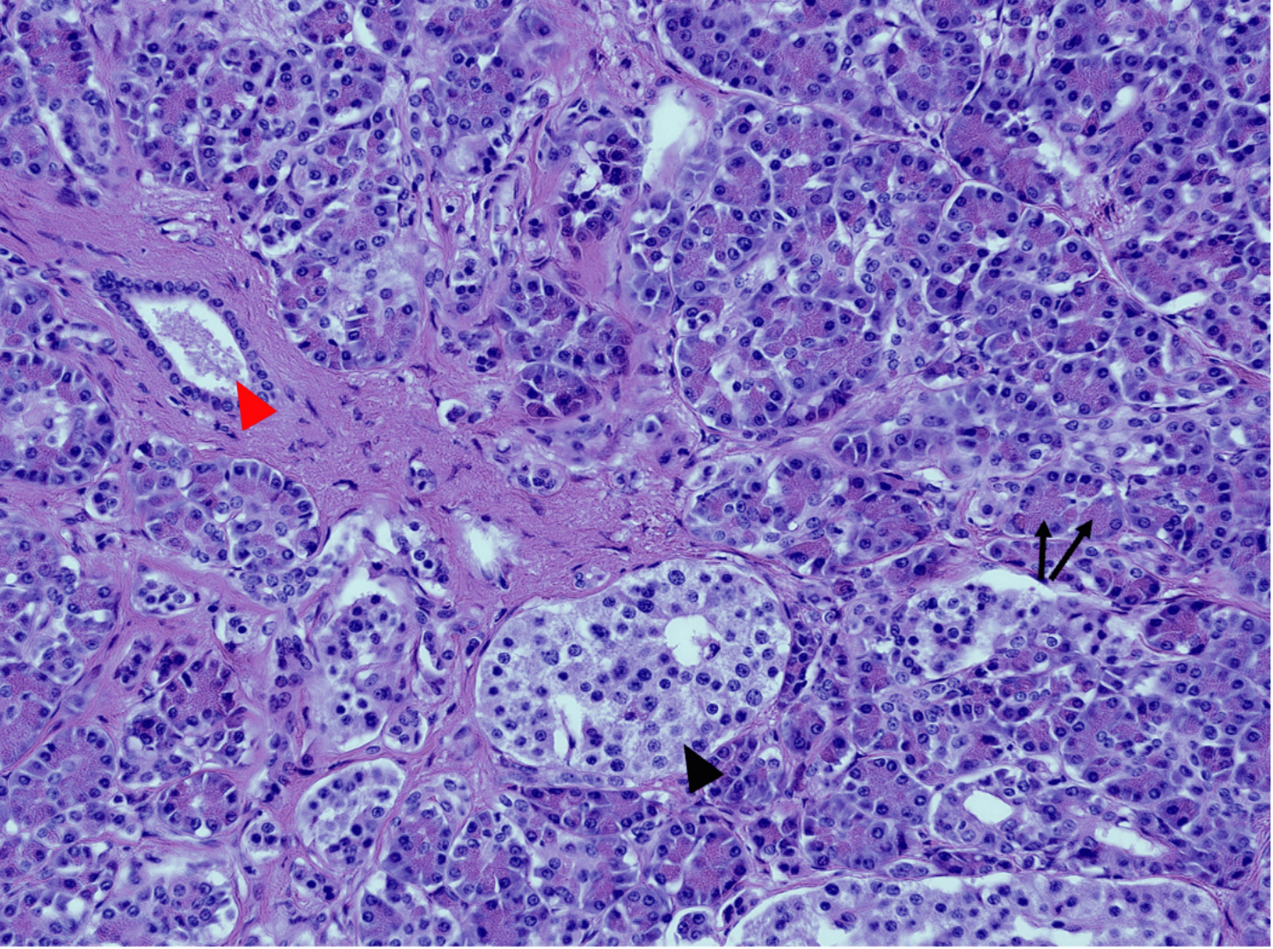
Resected ectopic pancreatic tissue composed of acini (black arrows), ducts (red arrowhead), and islets of Langerhans (black arrowhead) classifying the tissue as Heinrich type I (medium power (10x), H&E) H&E: hematoxylin and eosin

**Figure 7:**
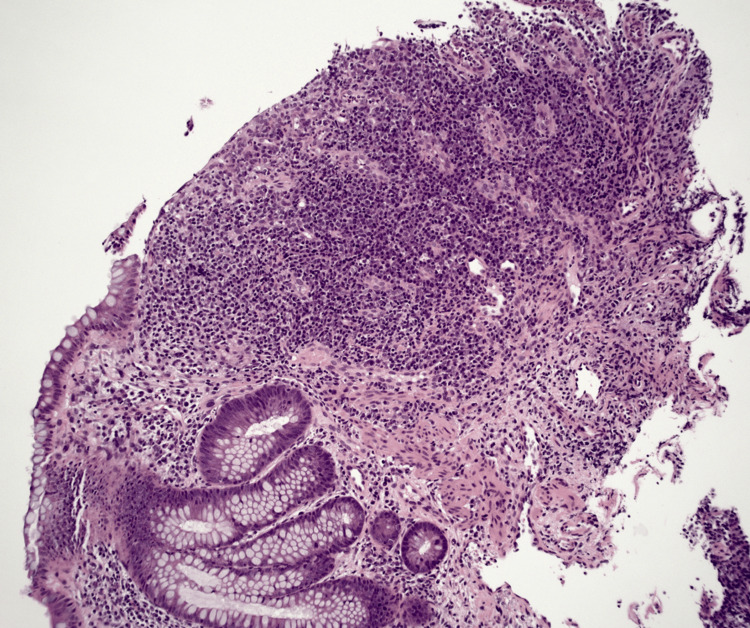
More pronounced changes indicative of chronic colitis including features of crypt loss and dense inflammatory infiltrate as well as crypt fusion (low power (4x), H&E) H&E: hematoxylin and eosin

**Figure 8:**
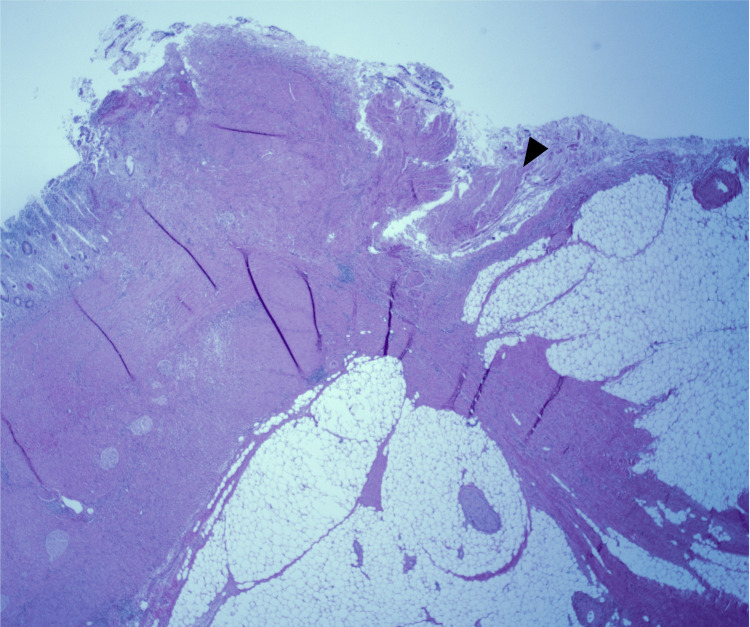
Colon with evidence of perforation as evident by full thickness loss of muscularis mucosa (black arrowhead) (low power (2x), H&E) H&E: hematoxylin and eosin

Several years after the initial diagnosis, she presented to the emergency department with abdominal pain, diarrhea, hypotension, fever, and tachycardia, meeting the criteria for systemic inflammatory response syndrome. After obtaining blood cultures, receiving fluids, and administering empiric antibiotics, imaging studies revealed a fistula between the previously strictured transverse colon and the small bowel at the duodenal/jejunal junction, causing colonic obstruction (Figure 4A-4B). She underwent a hemicolectomy and proximal jejunal resection to remove the fistulous segments of the bowel. Macroscopic examination of the jejunum showed a 2.8 x 2.8 x 2.2 cm yellow lobulated mass, insinuated between the fistulous portions of the large and small bowel (Figure 4A-4C). The ectopic tissue spanned from the jejunal wall into the pericolic fat (Figure 5A-5B). Histopathological evaluation of the mass revealed heterotopic pancreatic tissue with acinar glands, ducts, and islets consistent with Heinrich type I (Figure 6). The colon showed a fistula with adjacent areas of severe chronic active colitis and ulceration (Figures 7-8). Interestingly, the gallbladder was also removed, showing significant serosal inflammation. The patient was stabilized and discharged. She elected to discontinue treatment for IBD.

Despite the discontinuation of immunosuppressive therapy, the patient remained in remission after the resection of PH. A colonoscopy with biopsies two years after the resection showed no evidence of inflammation in the small bowel and colon, leading to a reevaluation of the Crohn’s disease diagnosis.

## Discussion

PH is a congenital anomaly involving the development of pancreatic tissue without anatomical or vascular connections to the pancreas [5]. Despite lacking a physical connection to the pancreas, ectopic tissue can possess histological features such as pancreatic acini, islets of Langerhans, and duct development [6]. Common locations for ectopic tissue include the duodenum (93.6%), stomach (24-38%), jejunum (0.5-27%), and Meckel’s diverticulum (2-6.5%) [5]. A classification of the histopathologic components of the ectopic tissue was developed by Heinrich in 1909 and modified by Gasper-Fuentes in 1973 [6]. This classification delineates four types of PH, each exhibiting variations in the microscopic components of pancreatic tissue. Type I describes typical pancreatic tissue with acini, ducts, and islet cells closely resembling the normal pancreas. Type II is the canalicular variety, consisting of ducts only. Type III is the exocrine type, composed of acinar tissue exclusively. Lastly, type IV is the endocrine type, characterized by the presence of islet cells alone.

The ectopic tissue from this patient was classified as type I, indicating its capability to produce pancreatic hormones and digestive enzymes and possess a functional ductal system for transporting these substances to the lumen of the bowel. While PH is usually asymptomatic, when symptomatic, it typically affects middle-aged patients and clinically manifests as abdominal pain, nausea, and diarrhea [6]. Despite advancements in diagnostic imaging and serological testing, the discovery of an ectopic pancreas is usually an unexpected finding, even in symptomatic cases, typically during surgical resection and histopathological evaluation. Surgical resection serves as a curative intervention, leading to the alleviation of symptoms.

Crohn’s disease is a chronic relapsing IBD, typically peaking in prevalence between the ages of 30 and 39. Its hallmark symptom is chronic diarrhea, defined as lasting more than four weeks. Other common presenting symptoms include abdominal pain (70%), weight loss (60%), and hematochezia, or the presence of mucus in stools (40-50%) [7]. Diagnostic procedures such as ileocolonoscopy and biopsy are recommended to confirm the diagnosis, often revealing characteristic findings such as a cobblestone-like appearance of the mucosal surface, skip lesions along the gastrointestinal tract, and the presence of fissures, fistulas, and strictures [3,8]. The mainstay of treatment for moderate to severe Crohn’s disease involves biologic therapy, sometimes combined with immunosuppressant therapy, and surgery may be necessary when indicated.

The patient's presentation closely resembled that of many individuals with autoimmune IBD but can be entirely explained by the presence of a pancreatic tissue mass in the jejunum. In ectopic tissue containing functional histological elements, especially acinar glands and a ductal system, symptoms often arise from local irritation and inflammation, facilitated by the secretion of digestive enzymes through the appropriate duct system. The ectopic tissue found in this patient featured a ductal network connecting a group of acini to the lamina propria of the small bowel mucosa and acini to the serosal layer, likely resulting in the secretion of caustic substances into the lumen of the jejunum as well as the peritoneum, respectively. Consequently, the patient experienced severe colitis and persistent peritonitis, leading to stricture formation, ulceration, and a fistula between the jejunum and the adjacent transverse colon. It is hypothesized that the formation of the anal fistula in this patient represents another instance of local change and inflammation resulting from the passage of caustic exocrine substances from ectopic pancreatic tissue through the gastrointestinal tract. The localized inflammatory disease in the anus, as opposed to other distal areas of the colon, may be due to the presence of a pre-existing mild anal condition, such as a fissure or ulcer, which became particularly sensitive to the caustic pancreatic substances.

Upon closer assessment of the patient’s clinical history and presentation, she was diagnosed with Crohn’s disease in her early 50s, which is later than the typical age for an initial diagnosis. Additionally, she denied any upper gastrointestinal symptoms associated with Crohn’s, as well as any typical extraintestinal manifestations, which can be observed in up to 33% of patients [7]. Notably, the terminal ileum was not involved in this patient, despite its involvement in 45% of Crohn’s cases [9]. Moreover, the patient lacked evidence of noncaseating granulomas, which are typically detected in 40-60% of surgically resected bowel segments in Crohn’s disease patients [10].

Furthermore, the non-healing surgical bed following the removal of her adrenal gland, causing saponification of fat and profound serosal inflammation of the gallbladder, may be attributed to pancreatic digestive enzymes. These caustic agents cause inflammation in the abdominal cavity that cannot be explained by other conditions, including IBD. The most significant evidence supporting the idea that PH caused symptoms resembling IBD is that, with the removal of the ectopic tissue from the jejunum and discontinuation of medical treatment for IBD, the patient made a full recovery. Subsequently, colonoscopy findings also revealed no evidence of inflammation along the gastrointestinal tract, further supporting the efficacy of curative surgery involving PH removal.

## Conclusions

This case illustrates the overlapping clinical presentation of symptomatic PH and IBD, potentially leading to misdiagnoses. Even when a patient’s presentation strongly resembles the diagnostic features of IBD, PH should be considered in the differential diagnosis. PH represents an anatomical cause of inflammatory small bowel and/or colonic disease, alongside autoimmune conditions like Crohn’s disease and ulcerative colitis. If PH is discovered, it is crucial to thoroughly document the anatomical and histological features of the ectopic tissue in the surgical pathology report. This documentation should include details such as the tissue’s size, the layers of bowel wall involved, the components of the pancreas present, and any areas showing ductal communication with the bowel lumen and adjacent tissues. Such features are essential for framing the patient’s prior symptoms, revisiting prior diagnoses, and planning further management.
